# Supplementary material for: Safety and health measures compliance during the COVID-19 pandemic for community-based tourism in Nakhon Si Thammarat, Thailand: A cross-sectional descriptive study
Source: PLoS One. 2024 Mar 5;19(3):e0300030. doi: 10.1371/journal.pone.0300030 (PMC10914287; doi:10.1371/journal.pone.0300030)
Supplement: Attachment — Submitted filename: Response reviewers - Final AB 01122023.docx [file pone.0300030.s001.docx]

**Response reviewers’ comments**

| **Date:** | Oct 18 2023 06:00PM |
| --- | --- |
| **To:** | "Apirak Bumyut" apirak.bu@mail.wu.ac.th |
| **From:** | "PLOS ONE" plosone@plos.org |
| **Subject:** | PLOS ONE Decision: Revision required [PONE-D-23-22531] |

PONE-D-23-22531

Safety and Health Measures Compliance during the COVID-19 Pandemic for Community-Based Tourism in Nakhon Si Thammarat, Thailand: A Cross-Sectional Descriptive Study

PLOS ONE

| **Reviewer** | **Comment** | **Response** |
| --- | --- | --- |
| 1 | The study deals with a very current issue, which is particularly prominent in the investigated area. The objective of the research is clear and well defined. At the same time, the scientific foundation of the research is less visible. I recommend a more prominent presentation of the literature review chapter in the study. The methodology of the research is relevant to the realization of the set goals; therefore I accept the results obtained. | Thank you for your comment, we agree with you. We added literature review chapter in the study. |
|  | At the same time, I recommend strengthening the visualization of the results, using figures instead of difficult-to-understand tables. Overall, a good quality study, minor revision is required.1 | We changed Table 4. to Fig 3.  Line: 294 |
| 2 | This study focuses on the level of knowledge and practices of entrepreneurs during the COVID-19 period, using a questionnaire survey as the research method. Unfortunately, the conclusions of this manuscript are commonplace and can be deduced through intuition without the need for research. For example, the author mentions that the low compliance rate among entrepreneurs is due to a lack of understanding and motivation to comply with standards, which is obvious. Therefore, I believe that this manuscript lacks a unique conclusion or viewpoint to support its publication, | Thank you for your valuable suggestion. We have added highlights on the barriers and support needed to assist CBT in complying with standards and building resilience in conclusion part.  Line: 438 - 444 |
|  | and the data from only 33 questionnaires is insufficient to support this conclusion. | 21 entrepreneurs participating in the study. This included 5 entrepreneurs from rural fishing communities (45.5% of the community's entrepreneurs), 7 from suburban orchard communities (87.5% of the community's entrepreneurs), and 9 from rural mountain communities (64.3% of the community's entrepreneurs) as showed in Study design.  Line: 185 - 188  However, the study has a limited number of participants because it took place during the COVID-19 pandemic in Thailand, causing the non-operation of the majority of CBT initiatives. Furthermore, specific establishments within the investigated CBTs were temporarily closed. As a result, the study was unable to recruit the participation of all establishments within the CBTs. Therefore, the result is specified for the three areas which not represent all CBTs in Nakhon-Si-Thammarat.  Line: 426 - 431 |
|  | Furthermore, this manuscript has some structural and logical flaws that need to be revised, as follows: The introduction section provides limited discussion on the logic and relationship of the content related to Thai community tourism, COVID-19, and the knowledge and practices of entrepreneurs. | In the introduction, we added information about negative impact of COVID-19 to Entrepreneurs in Thailand’s CBT “Entrepreneurs in Thailand’s CBT faced their biggest challenge due to the unexpected threat of COVID-19, leading to panic among local entrepreneurs because of lockdown uncertainty. The local government's restrictions, such as restaurant and bar closures, directly caused revenue losses and business closures, as confirmed by several entrepreneurs [10]”  Line: 68 -72  “Research on knowledge, practices, and adherence to COVID-19 mitigation measures through safety and health protocols in CBT establishments in Thailand has been limited. This limitation is particularly concerning, as it is essential to address this gap. The knowledge, attitude, and compliance levels of establishment owners are foundational elements for developing establishments that can meet the required standards and effectively contribute to public health and safety in the CBT context.”  Line 79 - 84 |
|  | The Ethics Statement is not suitable to appear in the Materials and Methods section; it should be included as formatted text at the end of the manuscript, before the references. | We have moved the ethics approval to the end of materials and methods section  Line: 222- 227 |
|  | Another flaw of this study is that the chosen research areas do not demonstrate uniqueness or universality. Why did the author select these three regions? Additionally, the author collected only 33 questionnaire responses from these three communities, and it is currently difficult to believe that these data are sufficient to support the conclusion. | CBT in three areas were operated during COVID-19 pandemic and recommended by Tourism Authority of Thailand (TAT) Nakhon Si Thammarat Office.  Line: 169 - 170  However, the study has a limited number of participants because it took place during the COVID-19 pandemic in Thailand, causing the non-operation of the majority of CBT initiatives. Furthermore, specific establishments within the investigated CBTs were temporarily closed. As a result, the study was unable to recruit the participation of all establishments within the CBTs. Therefore, the result is specified for the three areas which not represent all CBTs in Nakhon-Si-Thammarat.  Line: 426 - 431  For this study, 21 participants are represent ##% of the total population of entrepreneurs in study areas |
|  | The author should provide the content of the questionnaire in the supporting documents, as there seems to be a discrepancy between the number and structure of the questionnaire items and the amount of data collected.  The handling of questionnaire data in this study is also preliminary, with little consideration given to factor structure and relationships. | The Questionnaire in this study based on SHA standard. |
| 3 | The authors selected an actual but challenging topic for their research. Challenging, as the relation of community tourism and pandemic measures is controversial and hard to control the introduction and use of regulations. I regard the paper as original as both the approach and the selected methodology are unique and gap filling.  The structure and the execution of the research is acceptable. My main problem is that in fact there is no literature review in the manuscript, so it is quite a technical paper and hard to understand the context and the need for this particular research.  Some sources are mentioned in the Introduction chapter but is is far not enough. I recommend to write a separate literature review where the different international sources are critically analysed, e.g. <https://doi.org/10.21003/ea.V185-08> | Thank you very much for your useful recommendation. We added literature review chapter in the study which showed many studies about the concept of sustainable tourism and CBT from other countries as showed in literature review part.  Line: 93 – 165 |
|  | The implication of the research results are not indicated. | We added the implication of the research results at the last paragraph of discussion part following:  As a result, addressing barriers to compliance requires a holistic approach. Efforts should encompass comprehensive training, targeted education on specific measures, economic support from the government and other related organizations, and clear communication of the benefits of compliance. Tailoring initiatives to the unique challenges faced by small establishments in the CBT sector is crucial for fostering a resilient and compliant industry, particularly in the face of evolving challenges in the future such as the COVID-19 pandemic.  Line: 438 - 444 |
|  | The limitations of the research are not indicated. | We added the limitations of the study at the last paragraph of discussion part following: However, the study has a limited number of participants because it took place during the COVID-19 pandemic in Thailand, causing the non-operation of the majority of CBT initiatives. Furthermore, specific establishments within the investigated CBTs were temporarily closed. As a result, the study was unable to recruit the participation of all establishments within the CBTs. Therefore, the result is specified for the three areas which not represent all CBTs in Nakhon-Si-Thammarat.  Line: 426 - 431 |
| 4 | The topic of the paper is relevant and interesting, but very special. Authors used related literature sources and cited them correctly. Most part of them are from the last few years, but I suggest them restructure the paper and separated literature review and improve it with further connecting sources.  There were a lot of difficulties and new circumstances based on COVID-19. That was the reason that the purpose of the paper was to evaluate levels of knowledge, practice, and compliance regarding safety and health measures of the entrepreneur in managing CBT under the Safety and Health Administration (SHA) standard in the new normal situation.  In their paper they analysed 34 entrepreneurs from 3 subdistricts – (11) rural fishing-, (8) sub-urban orchard- and (14) rural mountain community – in Nakhon Si Tammarat province in 2021 between February and May. | Thank you so much, we appreciate your suggestion. We added literature review chapter in the study which showed many studies about sustainable CBT from other countries as showed in literature review part.  Line: 93 – 165 |
|  | They used correct methods which helped them to introduce their results.  Figures and tables are informative and increase the value of the paper.  I appreciate their results and conclusions which contain both theoretical and practical remark for the future. | Adjust resolution of figures in this study  We add the practical remark for the future. In the conclusion part following: As a result, addressing barriers to compliance requires a holistic approach. Efforts should encompass comprehensive training, targeted education on specific measures, economic support from the government and other related organizations, and clear communication of the benefits of compliance. Tailoring initiatives to the unique challenges faced by small establishments in the CBT sector is crucial for fostering a resilient and compliant industry, particularly in the face of evolving challenges in the future such as the COVID-19 pandemic.  Line: 438 - 444 |
