# Supplementary material for: Safety and health measures compliance during the COVID-19 pandemic for community-based tourism in Nakhon Si Thammarat, Thailand: A cross-sectional descriptive study
Source: PLoS One. 2024 Mar 5;19(3):e0300030. doi: 10.1371/journal.pone.0300030 (PMC10914287; doi:10.1371/journal.pone.0300030)
Supplement: Attachment — Submitted filename: renamed_2caec.docx [file pone.0300030.s002.docx]

**Response reviewers’ comments**

**PONE-D-23-22531R1**

**Safety and Health Measures Compliance during the COVID-19 Pandemic for Community-Based Tourism in Nakhon Si Thammarat, Thailand: A Cross-Sectional Descriptive Study**

| **Reviewer** | **Comment** | **Response** |
| --- | --- | --- |
| 5 | **Title:** I suggest shortening the current title. | Thank you for your feedback. While I appreciate your suggestion to shorten the title of my manuscript, "Safety and Health Measures Compliance during the COVID-19 Pandemic for Community-Based Tourism in Nakhon Si Thammarat, Thailand: A Cross-Sectional Descriptive Study," I respectfully prefer to retain the current title as it effectively captures the scope and focus of my research, which has already been considered from four reviewers. |
|  | **Abstract:** The abstract is well-written. However, I encourage you to remove the structured abstract format (background, methods, results, conclusion). Your abstract lacks a brief description of the methodology used, and consider formatting the results to interpretations for demystifying your findings. | Thank you for your recommendation. I agree with it, and consequently, I have removed the structured abstract format (background, methods, results, conclusion) from the manuscript in lines 23–41. Additionally, I have included more details to provide a brief description of the methodology in lines 26–32. |
|  | **Introduction:** To align with scientific norms and standards, I suggest restructuring the introduction section. It should include a general overview of the topic, relevant previous studies, a brief methodology description, brief findings, theoretical contributions, managerial implications, and a clear statement of the research gap you intend to address. The final paragraph should outline the paper structure. | Thank you for your valuable feedback. I appreciate your suggestion to restructure the introduction section to align with scientific norms and standards. I want to assure you that I have already incorporated the suggested elements in the introduction, including a relevant previous study (lines 76 - 79), a brief methodology description and standards that will be used the assess the compliance (lines 57 - 64), research gap (lines 68 – 71 and 79 - 84), and managerial implications (lines 90 - 92). Based on your recommendation, a brief methodology description was added in lines 86 – 88. |
|  | **Literature Review:** While the literature review is well-structured, I recommend adding additional studies highlighting different perspectives on the topic, especially research conducted in the ASEAN context. Many sentences and statements should be justified in this section. | Thank you for your valuable feedback. In response to your suggestion to include additional studies highlighting different perspectives on the topic, especially research conducted in the ASEAN context, we have incorporated three specific examples in the literature review to enhance the diversity of perspectives. These examples cover Community-Based Tourism (CBT) in Kampong Grangsil, Jambangan Village, Dampit District, Malang Regency, Indonesia (lines 112 – 115), CBT in Brunei (lines 153 – 156), and CBT in Marakau Village, Sabah, North of Borneo (lines 156 - 159). |
|  | **Methodology:** While your chosen research method aligns with the paper&#39;s objectives and is well-detailed, it lacks sufficient description and justification for the techniques used. | Thank you for your suggestion. We have addressed this concern by incorporating references to support the chosen methodology in lines 180 – 181. |
|  | **Results and Discussion:** The results are well-presented. In the discussion section, I recommend offering necessary comparisons with previous studies. | Thank you for your suggestion. We appreciate your positive assessment of the presentation of our results. In response to your suggestion, we have incorporated comparisons with previous studies in the discussion section. Specifically, we have included a comparison with the research on "Knowledge, attitudes, and practices towards COVID-19: A community survey in Southern Ethiopia," as suggested, in lines 349 - 353. Additionally, we have included references to other relevant previous research to support our discussions:  Lines 328 – 337: Positive perceptions about CBT development were influenced by capacity building and official training provided by government organizations to entrepreneurs in CBT.  Lines 364 – 368: Knowledge and education levels were not the sole factors influencing entrepreneurs' compliance with ecotourism measures.  Lines 405 – 409: The benefits gained from standards compliance for small establishments were challenging to justify in comparison with the costs incurred, especially when compared to larger establishments.  Lines 415 – 417: Increased awareness of risks among individuals and the effectiveness of protective measures have led to improved compliance with COVID-19 prevention measures.  These additional references provide a more comprehensive understanding of the factors influencing compliance and outcomes discussed in our research. |
|  | **Conclusion:** The conclusion should succinctly summarize the main contributions of the research in advancing knowledge in the field and the main implications. | Thank you for the valuable feedback. In response to your suggestion, I have ensured that the conclusion effectively summarizes the main contributions of the research in advancing knowledge within the field. This information can be found succinctly presented in lines 441 - 445. Additionally, the main implications of the study are summarized in lines 445 - 451. I believe this approach provides a clear and concise overview of the research's significance and its potential impact on the field. Your guidance has been instrumental in refining the conclusion for better clarity and focus. |
|  | **References:** Consider adding recent references, mainly from reputable journals Q1 and Q2. | Thank you for your recommendation. Your suggestion is essential for improving the quality of the manuscript. Therefore, I have included the original article in Q1, published on August 3, 2023, in Line 342. Furthermore, we aim to ensure the high quality of references in our manuscript by citing high-quality publications as follows: Out of the 62 references in the manuscript, which include 45 research articles, all the research articles in the reference section were categorized into 25 Q1 publications (55.6%) and 9 Q2 publications (20.0%). Additionally, 34 research articles (75.6%) were published between 2019 and 2024. |
